# Coronary Artery Spasm: From Physiopathology to Diagnosis

**DOI:** 10.3390/life15040597

**Published:** 2025-04-03

**Authors:** Ilinca Savulescu-Fiedler, Radu Octavian Baz, Radu Andrei Baz, Cristian Scheau, Andrei Gegiu

**Affiliations:** 1 Department of Internal Medicine, The “Carol Davila” University of Medicine and Pharmacy, 050474 Bucharest, Romania; 2 Department of Internal Medicine and Cardiology, Coltea Clinical Hospital, 030167 Bucharest, Romania; 3 Clinical Laboratory of Radiology and Medical Imaging, “Sf. Apostol Andrei” County Emergency Hospital, 900591 Constanta, Romania; 4 Department of Radiology and Medical Imaging, Faculty of Medicine, “Ovidius” University, 900527 Constanta, Romania; 5 Department of Physiology, The “Carol Davila” University of Medicine and Pharmacy, 050474 Bucharest, Romania; 6 Department of Radiology and Medical Imaging, “Foisor” Clinical Hospital of Orthopaedics, Traumatology and Osteoarticular TB, 030167 Bucharest, Romania

**Keywords:** coronary artery spasm, vasospastic angina, endothelial dysfunction, physiopathology, inflammation, ischemia, computed tomography angiography, autonomic nervous system

## Abstract

Coronary artery spasm (CAS) is a reversible vasoconstriction of normal or atherosclerotic epicardial coronary arteries with a subsequent reduction in myocardial blood flow, leading to myocardial ischemia, myocardial infarction, severe arrhythmias, or even sudden death. It is an entity that should be recognized based on a particular clinical presentation. Numerous differences exist between CAS and obstructive coronary disease in terms of mechanisms, risk factors, and therapeutic solutions. The gold standard for CAS diagnosis is represented by transitory and reversible occlusion of the coronary arteries at spasm provocation test, which consists of an intracoronary administration of Ach, ergonovine, or methylergonovine during angiography. The pathophysiology of CAS is not fully understood. However, the core of CAS is represented by vascular smooth muscle cell contraction, with a circadian pattern. The initiating event of this contraction may be represented by endothelial dysfunction, inflammation, or autonomic nervous system unbalance. Our study explores the intricate balance of these factors and their clinical relevance in the management of CAS.

## 1. Introduction

Coronary artery spasm (CAS) is defined as a reversible vasoconstriction occurring in normal or atherosclerotic epicardial coronary arteries that reduces blood flow through the myocardium, leading to myocardial ischemia. In a broad sense, coronary vasospasm refers not only to epicardial vessel spasm but also to small vessel vasospasm [1] that conducts a reduced blood supply to a localized myocardial area, in response to vasospasm. The consequences of coronary spasm depend on the amount of myocardial flow reduction, the duration of vasospasm, and the local coronary situation. If the myocardial flow reduction is very high, or the local conditions are particular, CAS may lead to acute myocardial infarction, severe arrhythmias, or even sudden death. If CAS does not last long, flow disturbance is reversible, but if prolonged, it leads to platelet activation and thrombus generation [2,3].

In contrast to classical angina induced by increased myocardial oxygen demand, vasospastic angina occurs at rest or during regular efforts. Patients may describe pain; nonetheless, silent ischemia is most prevalent, and ECG shows transient ST segment elevation or depression [4,5].

CAS diagnosis is based on clinical symptoms, the most frequent symptom being represented by angina pectoris. Angina pectoris usually appears at rest and occurs mainly from midnight until very early in the morning. The precipitating factors are numerous, *with* the most common being exposure to a cold environment [6], exposure to emotional stress, long-term mental stress [7], hyperventilation [8], alcohol consumption [9], exposure to psychoactive drugs like cocaine, marijuana, or amphetamine [10,11,12], and magnesium deficiency [13].

As it was initially described by Prinzmetal as “a variant form of angina pectoris” [14], CAS occurs mainly at rest, not when the oxygen demand is high, as in exercise, suggesting a different pathophysiologic mechanism from classical angina. This thesis is also supported by the circadian rhythm of ischemic events in CAS, which differs from the 24 h distribution of ischemic events in coronary artery disease (CAD). So, myocardial infarction reaches the highest frequency peak between 6 AM and noon, followed by another peak, later in the day, during 6–8 PM [15]. On the contrary, variant angina showed a different circadian pattern, with a frequency peak early in the morning, between 2 and 4 AM [16]. The asymmetric distribution of ischemic events during the day is linked to the circadian variations in various neurohumoral factors. In the morning, α-sympathetic activity [17] and cortisol plasma levels are increased, and, as a consequence of the epicardial vessel’s sensitivity to vasoconstrictor stimuli, the vascular tone also increases [18].

CAS has seasonal variation, and the coronary events due to CAS mostly occur in winter and spring [19], as in obstructive coronary artery disease [20,21].

At the beginning of the coronary spasm, especially if it is a mild one, a standard 12-lead electrocardiogram (ECG) may be normal, but with spasm progression, ECG recordings may show ST-segment or T-wave changes in the electrocardiogram leads, corresponding to the territory of the vasospastic coronary artery: ST-segment elevation, in cases of subtotal or total spasm of a major artery [22], or ST-depression in myocardial ischemia, caused by occlusions of small arteries or if a major artery that receives collaterals is occluded [23]. In focal proximal coronary spasm ECG may reveal symmetrical T waves and ST-segment changes [24].

Besides the ECG changes that suggest myocardial ischemia, various types of arrhythmias can occur during CAS, including supraventricular tachyarrhythmias, asystole, malignant ventricular arrhythmias (ventricular tachycardia and/or fibrillation, mostly when the left main or left anterior descending coronary artery are involved), and AV block, in cases of inferior ischemia. Sudden death related to CAS often results from bradyarrhythmia rather than from tachyarrhythmia [25,26].

Other non-invasive, non-pharmacological methods to diagnose the CAS except ECG include continuous Holter monitoring and exercise testing. These techniques align with current clinical guidelines [27].

## 2. Imaging Approach of Coronary Artery Spasm

The gold standard diagnostic tool for CAS is represented by a transient subtotal or complete, but reversible, occlusion of coronary arteries, with more than 90% transient vasoconstriction of the coronary artery at spasm provocation test, which consists of an intracoronary administration of Ach, ergonovine, or methylergonovine [28] during angiography. During the procedure, Ach dissolved in saline is administered in the left coronary artery in increasing doses ranging from 20 to 200 µg to assess vasoreactivity over 20 s, and the angiography is performed one minute later [29]. The test can also be performed in the right coronary artery to ensure a comprehensive evaluation of coronary vasomotor function, with doses ranging from 20 to 80 µg [30,31,32]. Patients are advised to discontinue medications such as nitrates, calcium channel blockers, beta-blockers, and other vasodilators or vasoconstrictors at least 72 h prior to the procedure to avoid interference with the results of the test. Continuous monitoring of the patient’s ECG and blood pressure is essential throughout the test to detect ischemic changes and ensure safety. If significant spasm occurs, or if the patient experiences severe symptoms or significant ECG changes, the infusion is stopped immediately and the spasm is reversed by administering an intracoronary injection of nitroglycerin [30,31].

During this procedure, some complications may occur which include angina, dyspnea, vomiting, and arrhythmias. Intracoronary provocation testing should not be performed in patients without symptoms of vasospastic angina (VSA), pregnant women, or in patients with severe hypertension, significant left main artery stenosis, advanced heart failure, severe aortic stenosis, or renal failure (creatinine > 2.0 mg/dL), or severe asthma [30,31].

The intracoronary acetylcholine provocation test is globally underutilized, primarily due to concerns about potential complications, especially in acute clinical situations, ultimately leading to inappropriate therapeutic choices [33].

Scoring systems are essential in the diagnosis of CAS, offering predictions based on a variety of clinical, paraclinical, and imaging risk factors. This allows for a risk-based patient stratification, improving diagnostic accuracy and reducing the need for unnecessary testing and procedures. A particularly interesting approach in this regard is the ABCD score proposed by Rinaldi et al., which determined three clinical predictors and one angiographic predictor of a positive provocation test response [34].

Historically, other non-invasive methods such as hyperventilation, the cold pressor test, or ergonovine echocardiography have been used to assess CAS. While the first two have low diagnostic accuracy, the latter can pose risks, as it may induce persistent multi-vessel coronary spasm that does not respond to sublingual or intravenous nitroglycerin [35,36].

Over time, studies have linked CAS to several biomarkers, including C-reactive protein, soluble CD40 ligand, plasma xanthine oxidoreductase activity, cystatin C, Rho-kinase activity in circulating leukocytes (for epicardial CAS), and serotonin (for microvascular CAS). However, apart from increased Rho-kinase activity in circulating leukocytes, no reliable biomarker for CAS diagnosis has been identified to date [37,38,39,40,41].

Other diagnostic tools are represented by optical coherence tomography (OCT) and intravascular ultrasound (IVUS) [42]. Intracoronary imaging provides not only morphological changes in coronary vessels but also the association between the atherosclerotic plaque and the vasospasm, if existent [43]. Further, intracoronary imaging is not a routine examination because of its complexity. OCT enables intima estimation; meanwhile, IVUS, which has a deeper penetration, interrogates the perivascular injury [44].

CCT is valuable for ruling out significant coronary artery disease or stenosis that could either mimic or contribute to CAS. While it effectively excludes obstructive coronary disease [28], its ability to detect subtle plaque rupture or erosion, both of which are key factors in spontaneous coronary artery dissection and acute coronary syndromes, is limited. Interestingly, CCT may reveal coronary stenosis, which may later be identified as vasospasm upon further invasive coronary angiography.

Dual-acquisition cardiac CT involves obtaining a baseline CCT scan without vasodilators, followed by a second scan with intravenous nitrate infusion, typically performed within two weeks. It has shown potential for identifying vasospastic angina, with high specificity and positive predictive value. However, sensitivity remains suboptimal, requiring further refinement towards the improvement of its diagnostic accuracy [45].

Cardiac MRI plays a crucial role in detecting myocardial abnormalities, including edema, fibrosis, and scarring. Late gadolinium enhancement (LGE) in the subendocardial layer is indicative of ischemic injury related to plaque rupture, vasospasm, thromboembolism, or dissection, while subepicardial LGE suggests myocarditis or cardiomyopathy.

Evaluation of the perivascular tissue inflammation that surrounds coronary arteries is performed at PET/CT [29].

Echocardiography should be performed acutely to detect regional wall motion abnormalities, which may indicate myocardial ischemia or infarction. Additionally, it can help identify pericardial effusion, a potential complication of coronary vasospasm or ischemic injury [46,47].

The main aspects of diagnostic modalities for CAS are summarized in Table 1.

## 3. Risk Factors for Coronary Artery Spasm

The main risk factors are the classical risk factors for coronary artery disease (CAD), including age, smoking status, high LDL cholesterol levels, arterial hypertension, diabetes mellitus, and elevated hs-CRP [48,49,50,51]. However, LDL-cholesterol levels do not play a major role as a risk factor in CAS compared to coronary atherosclerosis, suggesting that the pathogenesis of these two entities may differ [52]. Nevertheless, oxidized LDL may activate the RhoA/RhoK pathway, which plays an instrumental role in the pathogenesis of CAS alongside inflammation, hypoxia, reactive oxygen species, and chronic stress [53,54,55,56]. A special relationship between hs-CRP levels and CAS has been shown. In patients with low levels of hs-CRP, the risk for CAS is associated with diabetes mellitus in men [48]; meanwhile, in patients (especially in women) with high levels of hs-CRP, the risk for CAS is negatively associated with diabetes mellitus and arterial hypertension [48]. Regarding the lipid profile, it is worth mentioning that while LDLc levels represent a risk factor (and a target for therapy) for CAD, in CAS, lipoprotein (a) plasma levels correlate better with CAS prognosis.

Although there are controversies regarding the contribution of lipoprotein (a) to the occurrence of CAS, many authors support the existence of a relationship between CAS and lipoprotein (a). Serum lipoprotein (a) levels are higher in patients with CAS compared to those without CAS [57], and also in patients with higher basal tone at the spastic site of coronary arteries [58]. Some authors reported a correlation between lipoprotein (a) concentration and the vasoconstrictor response to Ach [59]. In addition, lipoprotein (a) is involved in thrombotic coronary occlusion [60].

Some authors suggest that high cystatin C levels are independently associated with CAS occurrence, highlighting a potential role for cystatin C as a biomarker of CAS [40,61].

Magnesium is considered an endogenous calcium antagonist [52]; magnesium deficiency causes CAS, through decreased calcium channel blockage [52,62], with increasing intracellular calcium levels and calcium sensitivity of muscle cells. Magnesium deficiency is observed in 45% of patients with CAS [13]. In patients with low magnesium levels, magnesium supplementation must be performed [11].

It is important to mention that cigarette use is a striking risk factor for CAS occurrence, and three-quarters of patients with CAS are smokers [63]. Chronic exposure to particulate matter was also shown to correlate with epicardial spasm, higher chances of positive provocation test, and higher risk for myocardial infarction with no obstructive coronary arteries (MINOCA) [64,65,66].

Psychological stress is one of the recognized risk factors for ischemia in non-obstructive coronary artery disease. During mental stress, normally, in response to oxygen demand, epicardial vessels and microvessels dilate. But in ischemic heart disease, irrespective of obstructive or non-obstructive forms, epicardial vessels constrict [67,68]. Notably, the prevalence of anxiety and depression is higher in patients with CAS than in those with obstructive CAD [69]. Psychosocial stress effects may be linked to autonomic nervous system dysfunction [70].

Some genetic polymorphisms or mutations in genes coding angiotensin-converting enzyme [71], paraoxonase I [72], adrenergic receptors [73], endothelial nitric oxide (NO) synthase [74,75], serotoninergic receptors [76], and inflammatory mediators [77] have a contribution to CAS physiopathology.

A mutation in the ALDH2 gene is involved in CAS occurrence. The mutation in the ALDH2 gene, recognized mostly among East Asians [78], is responsible for decreased ALDH2 capacity to metabolize acetaldehyde [79]. Carriers of the ALDH2 mutation have a 48% higher risk for CAD, as compared with the population without this mutation [80]. This mutation is particularly important concerning alcohol consumption because the metabolic chain is interrupted at acetaldehyde, a free radical inducer [81], because of ALDH2 deficiency [79,80,81]. People who are deficient in ALDH2 are at higher risk of not only liver cirrhosis and gastric and esophageal cancer but also of CAD [78,79]. Further, the deficit variant ALDH2 genotype is rare in general populations, except in East Asians, in whom the prevalence is around 30–50% [82].

There are wide differences in the prevalence of CAS across different countries and among races [11]. CAS appears to have a greater prevalence in Japan and East Asia compared to Western countries, being more frequent in men between 40 and 70 y.o. and in women [22,83]. Further, the percentage of female and young people is higher than in obstructive coronary disease. Furthermore, the COVADIS study showed that ethnic differences in terms of prognosis or coronary reactivity are not relevant [84].

The epidemiological data are different in obstructive CAD and CAS. CAS more frequently affects younger patients and is more frequent in females than in males [85]. CAS is associated with smoking, cocaine, marijuana, and alcohol consumption, which at least partially explains the incidence of myocardial infarction in young people, without other significant risk factors [85]. One study explored the different prognostic significance of Ach provocative testing in patients with ischemia or myocardial infarction with non-obstructive coronary arteries [86]. This study, which included 519 patients followed up for 60 months, concluded that microvascular spasm has a higher incidence in females; a positive Ach was an independent predictor for major adverse cardiovascular and cerebrovascular events in men but not in women. Another conclusion of the study, however, was that the clinical outcomes of patients with myocardial ischemia or myocardial infarction with non-obstructive coronary artery did not differ between men and women with a negative or a positive ACh test [86].

CAS prevalence declines with age; meanwhile, atherosclerosis prevalence has an opposite evolution, increasing with age [52].

## 4. Normal Coronary Endothelium

From the lumen outward, the coronary artery wall consists of the tunica intima, tunica media, and adventitia.

The tunica intima of coronary arteries consists of the endothelial layer, the subendothelial space, and the internal elastic membrane. The endothelium is formed by a monolayer of endothelial cells (EC). This layer is not only a semipermeable barrier controlling solutes and extravasation but also has many specific functions, such as regulating coagulation, vascular tone, inflammation, and angiogenesis [87,88,89]. The subendothelial layer is represented mainly by vascular smooth muscle cells (VSMCs) and the extracellular matrix (ECM) [90]. The VSMCs proliferate at very low rates [91,92], but proatherogenic stimuli can determine their proliferation and production of large quantities of altered ECM [93].

The tunica media consists of several layers of VSMCs and ECM [94,95]. VSMCs have contractile and synthetic functions [94]. They regulate vessel diameter and blood flow in response to Ach or norepinephrine [96]. VSMCs are characterized by phenotypic plasticity [97], so in particular conditions, VSMCs change their phenotype from a contractile to a synthetic one [98].

The adventitia is represented mainly by connective tissue which includes collagen and elastic fibers, vasa vasorum, adrenergic nerves, and lymphatic vessels [96,99]. The fibroblasts, which represent the main cells in adventitial tissue, may proliferate and differentiate into myofibroblasts in response to stress or injuries [100], regulating EC and VSMC growth [101].

The vessels are surrounded by a special cellular population, represented by perivascular adipose tissue (PVAT) [102], in terms of a phenotype closer to brown adipose tissue. PVAT releases adiponectin, with vasodilator properties, alongside vasoactive factors that increase vascular contractility [102].

The endothelium is an anti-thrombotic surface, as a consequence of the synthesis of inhibitors of tissue factor and thrombin, and endothelial protein C receptors (EPCRs) contribute to protein C activation [103]. The mitochondrial thioredoxin system, which prevents ROS generation, also exhibits antithrombotic properties, as long as the loss of thioredoxin reductase 2 results in a prothrombotic phenotype of the EC [104]. In contrast, the endothelium releases von Willebrand factor, a pro-coagulant factor.

ECs release vasodilatory factors in response to mechanical stimuli, such as shear stress, and chemical stimuli, e.g., acetylcholine (Ach), angiotensin II, arachidonic acid, bradykinin, serotonin, and histamine, among others [105]. The main vasodilators produced by the ECs are NO (especially in large arteries) [56], the endothelium-derived hyperpolarization factor (EDHF) (especially in resistance arteries) [56], and prostacyclin; all these endothelium-derived relaxing factors are not only released in response to the stimulation but are also involved in resting vascular tone [106].

ECs also release vasoconstrictors, with the main vasoconstrictors endothelium-derived represented by thromboxane A2 (TXA2), endothelin-1 (ET-1), prostaglandin H2, and ROS [107,108].

In healthy individuals, an intracoronary infusion of Ach is followed by coronary vasodilatation because Ach induces NO release from the endothelium. This is not the case in subjects with coronary atherosclerosis, where Ach causes vasoconstriction [88,109,110]. Because of the high sensitivity of coronary arteries in patients with CAS, the intracoronary administration of Ach is used as a provocative test [14,111,112], and other provocative tests are represented by the administration of other vasoactive substances that induce the endothelial release of NO, such as bradykinin, histamine, and ergonovine [52]. The same vasoconstrictor effect as Ach was proved experimentally in pigs with mild atherosclerotic lesions upon the intracoronary administration of serotonin or histamine [113,114,115].

Serotonin acts as a vasodilator in normal coronary arteries, while exhibiting potent vasoconstrictive properties in endothelial dysfunction [116,117]. One study showed that serotonin levels are significantly higher in patients with CAS without coronary obstruction [118].

Neuropeptide Y is released after sympathetic stimulation and has vasoconstrictor effects, mainly at small vessel levels than in epicardial arteries [119,120].

Nitroglycerin administration promoting endothelium-independent vasodilation leads to super-sensitivity reactions in coronary arteries in patients with CAS [52].

A decrease in the vasodilator response functionally characterizes endothelial dysfunction, with the endothelial surface becoming prothrombotic and pro-inflammatory. Cardiovascular risk factors contribute to increased CRP, IL-1, and IL-6 production, which switch the endothelial phenotype toward a pro-inflammatory one [121].

## 5. Coronary Spasm and Coronary Atherosclerosis—Coronary Spasm and Thrombosis

Unlike small vessel (coronary) atherosclerosis, where the abnormalities in LDL cholesterol metabolism, diabetes mellitus, age, and smoking are striking risk factors, in CAS, the most significant risk factors are represented by chronic low-grade inflammation (hs-CRP), age, smoking, and remnant lipoproteins [122,123,124]. Arterial hypertension, a classical major risk factor for atherosclerosis and CAD, is negatively associated with VSA [48].

Around 70% of patients with angina show no obstruction of the coronary artery on coronary angiography. Still, despite this, they have myocardial ischemia [125].

Coronary spasm may be focal, diffuse, affecting one or more vessels, mixed (one coronary artery with focal spasm and another showing diffuse spasm), or migratory [85]. It is important to mention that patients who show diffuse spasms have a worse prognosis and are less likely to respond to medication [126].

A few OCT studies have shown that in patients with vasospasm-induced ACS, intimal erosion and intraluminal thrombi were found in most cases. Lerman et al. observed in their study [127] that coronary spasm was associated with vascular intimal injury, disruption, and erosion. Histological analysis of the endothelium changes, such as hyperplasia, thrombus formation, and hemorrhage found in patients with CAS, creates a strong link between spasm and plaque progression.

However, comparing vasospastic lesions to ACS lesions, there are different characteristics of the plaques using virtual histology IVUS, such as lower volume, smaller necrotic core, and dense calcium in the vasospastic lesions compared with culprit atherosclerotic lesions [127].

Intimal erosion and irregularity are the main findings in vasospastic lesions, while in coronary atherosclerosis, macrophages and intraplaque neo-vessels were observed using OCT in the segments with endothelial dysfunction. This finding reflects the role of inflammation and neovascularization in plaque progression and impaired vascular reactivity [128].

The distinction between ischemic events caused by coronary spasms and those caused by obstructive coronary artery disease is important from several perspectives: pathophysiological, diagnostic, and therapeutic. Myocardial ischemia can result from coronary obstruction or coronary spasm (non-obstructive ischemia). The two conditions may or may not coexist. CAS occurs in individuals with angiographically normal coronary arteries but is also described in patients with coronary atherosclerotic lesions. The coexistence of coronary lesions, even non-obstructive ones, with coronary spasms is associated with a worse prognosis [126]. Moreover, the co-existence of severe coronary artery disease with CAS is associated with a higher risk of severe myocardial injury [85,129]. However, the spasm preferentially occurs at bifurcations and in other locations than atherosclerotic lesions [5].

It is difficult to demonstrate whether the plaque itself induces coronary spasm, and angiography has shown that areas with spasm were parallel to atherosclerotic plaque [130,131]. The fact that coronary spasms can induce the rupture of a stable plaque is demonstrated and well-known [85].

### 5.1. Microvascular Dysfunction

Microvascular dysfunction is defined by an abnormal reduction in the microvascular wall at vasodilator stimuli and a hyperreactive response to vasoconstrictor stimuli. It plays a role in the pathophysiology of ischemia with no obstructive coronary arteries (INOCA). Predictors for microvascular dysfunction are female gender, minor ischemic ECG findings, reduced adenosine triphosphate-induced coronary flow reserve (ATP-CFR), and low body mass index [11,132].

The slow coronary flow phenomenon (CSFP) appears secondary to the spasm of the small coronary arteries, leading to low flow rates in the epicardial coronary arteries and facilitating endothelial platelet interaction. Structural remodeling of the microvasculature can occur due to the erosion of the glycocalyx caused by inflammation, which is initiated by platelets, mast cells, and the activation of neutrophils [133]. It was shown that CSFP is reversible, and symptoms are controlled better after treatment with mibefradil (a selective blocker of T-type microvascular calcium channels); this shows that CSFP is a form of microvascular CAS [133].

### 5.2. Spasm and Atherosclerosis

INOCA includes patients with no obstructive coronary artery disease (examined angiographically) but with evidence of ischemia. INOCA has shown an increasing prevalence over the past decade [134]. In patients with INOCA examined angiographically with coronary reactivity testing included, the majority (around 75–90%) received a diagnosis of coronary spasm, microvascular or epicardial spasm, coronary microvascular dysfunction, or myocardial bridging [135]. A study assessing the prognostic impact of COVADIS criteria in patients with INOCA and MINOCA undergoing ACh provocation testing revealed that the presence and number of positive COVADIS criteria correlate with higher rates of major adverse cardiovascular and cerebrovascular events, therefore suggesting that a thorough evaluation of these criteria yields a better prognostic stratification than a simple positive or negative classification [136].

Coronary spasms can often be observed in angiographically normal arteries but can also be found in patients with mild or more severe coronary artery disease; most of the time, these two entities coexist. So, the questions that need to be answered are as follows: in this case, does spasm cause angina? Does the presence of the plaque cause the spasm? And if a patient presents with both, do they have a worse prognosis? [85]. In a prospective registry, Jo et al. showed that patients with INOCA and coexistent vasospasm had more atherosclerotic lesions in all coronary arteries when compared with the ones without spasm. Further, one important thing to mention in patients with INOCA and CAS is that it was more likely to observe atherosclerosis in the spastic arteries [137], and this was even higher in the patients with focal CAS rather than in those with diffuse CAS. Therefore, atherosclerotic treatment may benefit patients with CAS and coronary atherosclerotic disease [138]. Regarding the interventional treatment of atherosclerotic lesions (PCI), recent studies have shown that PCI did not influence the recurrence of CAS, with the spasm being present diffusely in the distal segments of the stent [52].

### 5.3. Myocardial Bridging

Myocardial bridging (MB) is a congenital anomaly characterized by an epicardial coronary artery segment running within the myocardium, leading to dynamic systolic compression [139]. The left anterior descending (LAD) artery is the most frequently affected region.

**Figure 1:**
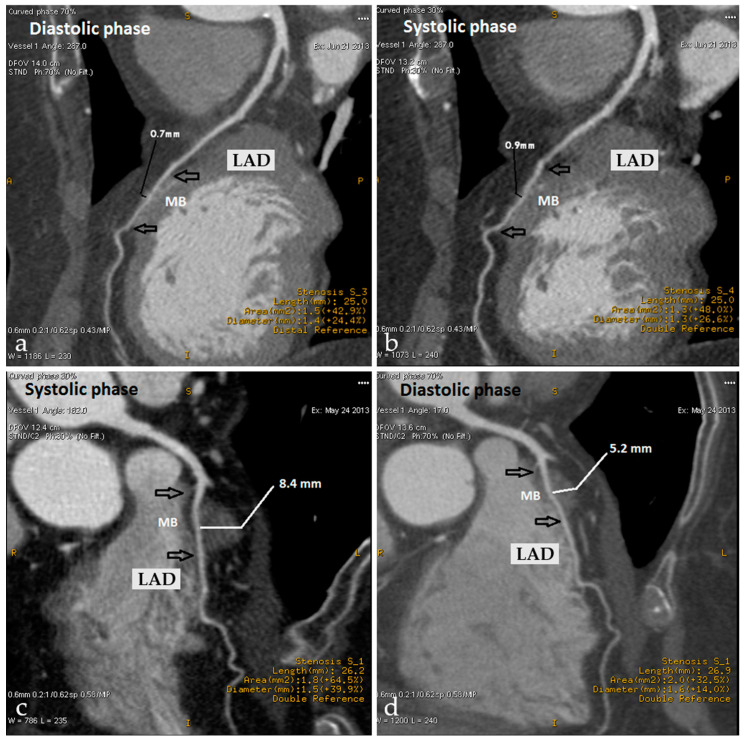
Cardiac computed tomography images of mid-diastolic (**a**) and end-systolic (**b**) phases showing very thin MB at middle LAD without significant systolic compression (arrows). Significantly thicker MB at middle LAD (**c**,**d**), demonstrating systolic compression of the tunneled artery (arrows).

CCT allows for a detailed anatomical assessment of MB, including its precise location, number, length, and depth [140]. It also enables functional evaluation by measuring the systolic compression effect on the tunneled arterial segment. The degree of luminal narrowing depends on MB thickness and depth, with more severe compression observed in deeper or thicker bridges [141] (Figure 1).

The hemodynamic impact of MB is influenced by multiple factors, including bridge length, depth, orientation, and the presence of coexisting atherosclerosis. Although MB has traditionally been regarded as a benign variant, evidence suggests that certain morphological features—such as deeper or longer bridges—can significantly impair diastolic coronary perfusion, contributing to myocardial ischemia [142]. While MB leads to a decrease in the diameter of the involved vessel, it is necessary to differentiate between MB, where the compression is extrinsic to the vessel, and CAS, in which there is increased contraction of the VSMC.

Advanced invasive imaging techniques such as intravascular ultrasound (IVUS) and fractional flow reserve (FFR) have revealed increased atherosclerotic burden proximal to MB segments, as well as altered coronary hemodynamics due to retrograde blood flow effects [139,141]. Moreover, stress perfusion cardiac MRI and computational fluid dynamics modeling have provided further insights into MB’s ischemic potential, underscoring the need for individualized risk assessment [140].

Given that coronary perfusion predominantly occurs during diastole, MB has historically been perceived as a benign anatomical feature [139,143]. However, mounting evidence suggests associations between MB and angina, acute coronary syndrome, and even sudden cardiac death [139,143]. The mechanisms underlying MB-induced ischemia are multifaceted, involving heart rate-dependent diastolic perfusion limitations, transmural perfusion imbalances, localized and systemic coronary artery disease, and coronary vasoconstriction [144,145,146].

Additionally, the repetitive compression–relaxation cycles exerted by MB may promote endothelial dysfunction and enhance vascular responsiveness to vasoconstrictor stimuli [147]. Research suggests that patients with MB and non-obstructive coronary artery disease exhibit a higher incidence of epicardial spasm when subjected to intracoronary provocative testing compared to individuals without MB [148]. Furthermore, both epicardial and microvascular coronary vasospasm have been implicated in the pathogenesis of MINOCA [149,150]. In patients with non-obstructive coronary artery disease, MB-associated vasospasm has been linked to more severe clinical manifestations, a heightened risk of MINOCA, and an increased rate of angina-related hospitalizations during long-term follow-up, despite a relatively low incidence of major adverse cardiovascular events [151].

CAS may play a significant role in modulating the clinical outcomes of patients with MB, potentially exacerbating myocardial ischemia and contributing to adverse cardiovascular events. While current evidence highlights a complex interplay between these two conditions, further large-scale, multicenter, and long-term prospective studies are essential to establish their precise relationship and optimize risk stratification and management strategies [152]. A deeper understanding of this association could improve the diagnostic and therapeutic approaches tailored to patients with CAS and MB, ultimately enhancing clinical outcomes.

The relationship between coronary artery bridging and CAS is complex and involves a variety of concurring factors including external compression, endothelial and microvascular dysfunction, and autonomic dysregulation [153]. Repeated compressions of the coronary in the systolic phase may lead to endothelial injury and alter normal vasodilatory mechanisms and nitric oxide production; moreover, the increased sympathetic activity due to stress or various pharmacologic factors can amplify the vasoconstriction, which is even more severe in patients predisposed to exaggerated vasomotor responses [154]. Understanding the interplay of these factors can lead to a better approach of patients with MINOCA, where myocardial bridging may be overlooked despite representing an essential factor [66,149].

### 5.4. Spasm and Coronary Thrombosis

It is known that one cause of acute coronary syndromes is represented by coronary thrombosis. It has been shown that after an attack of CAS, the plasma levels of fibrinopeptide A are increased [52], with no changes after an attack of stable exertional angina, leading to thrombin generation and the formation of thrombi in the involved coronary artery [155]. A circadian variation in the plasma levels of fibrinopeptide A has been observed in parallel with CAS. The same circadian variation has been noted in plasminogen activator inhibitor 1 levels. After a CAS attack, activation of the platelets has been observed, compared with a stable effort angina attack where this process happens at a smaller scale [52]. Prolonged CAS leads to coronary flow limitation and can favor thrombus formation without plaque rupture [156].

## 6. Pathophysiology of CAS

The pathophysiology of CAS is not fully understood. There is no consensus regarding the primary determining factor of CAS or the site of initiation of the pathophysiological cascade in CAS. However, at the center of the coronary spasm phenomenon lies the contraction of VSMCs with its circadian pattern. Rho-kinase, an enzyme proved to be implied in VSMC contraction, has circadian activity, so Rho-kinase shows higher activity during midnight and early in the morning [157,158].

One hypothesis is that VSMC contraction is triggered by endothelial dysfunction, a hypothesis strongly sustained by the paradoxical constriction in response to Ach. A questionable problem regarding endothelial dysfunction’s contribution to CAS is that the coronary spasm has a relative circadian rhythm; meanwhile, the endothelial dysfunction is practical continuously.

Inflammation is another factor whose contribution to CAS is considered “primum movens” in patients with vasospastic angina. In patients with SARSCoV2 infection, a disease characterized by cytokine storm, many severe cases of CAS were reported, but it is not certain the amount of cytokines responsible, if neural disorders are involved, or if they had CAS before COVID-19 infection [159,160].

One experimental study on pigs established a model of atherosclerosis and endothelial dysfunction, obtained through balloon endothelial removal in pigs exposed to high-cholesterol diets. The authors reported coronary spasms at mild atherosclerotic lesions after intracoronary administration of serotonin or histamine [113,114,115].

Another experimental model, also in pigs, had as its main objective the study of the link between coronary inflammation and coronary spasm, starting from the observation of the existence of a high-inflammatory infiltrate within the adventitia of spastic coronary segments [161]. The analyzed experimental model was conducted on pigs in which the coronary endothelium was preserved, but adventitial inflammation was induced through the local administration of IL-1b for 4 weeks [56,162,163,164,165]. In these subjects, the development of mild atherosclerotic lesions was observed, in areas in which VSMC hypercontraction was noted. At spastic coronary segments, the degree of MLC phosphorylation increased between the vasoconstrictor response to serotonin, and the MLC phosphorylation degree was a concordant relation [166]. Inflammatory stimuli such as IL-1b induce the up=regulation of Rho-kinase expression in VSMCs, as demonstrated in vitro [167].

In both experiments, the vasoconstrictor response was observed, in the first situation preceded by endothelial denudation and in the second model related to inflammation.

In conclusion, the experimental findings reinforce the notion that CAS is a complex disorder where inflammatory, endothelial, and platelet-mediated factors intertwine. The direct implications for patient care reside in the potential for developing new anti-inflammatory and antiplatelet treatments as well as targeted modulators of the vascular smooth muscle. Future studies should aim to validate these findings in human cohorts and explore novel therapeutic interventions that can provide better results compared to the classical vasodilator therapy.

**Figure 2:**
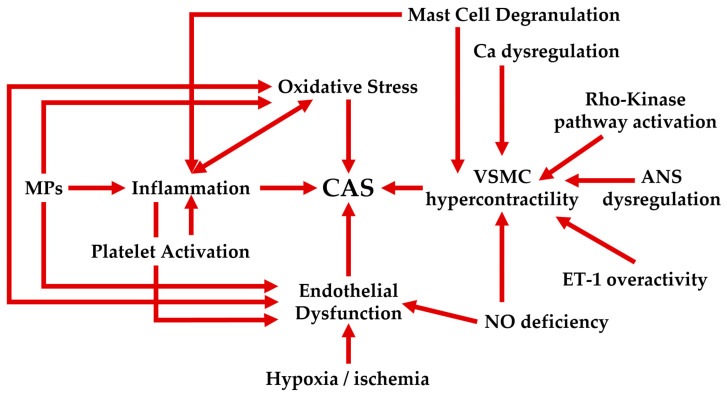
Vascular smooth muscle cells contraction is the core of the coronary artery spasm. VSMC = vascular smooth muscle cells, ANS = autonomic nervous system, MPs = microparticles, NO = nitric oxide; ET-1 = endothelin-1; Ca = calcium.

However, despite great progress on in vitro and animal models, the mechanisms that ground CAS are not completely clear. As definite, CAS is a multifactorial disease [62] that involves endothelial dysfunction, oxidative stress, VSMC hyper-reactivity, chronic inflammation, autonomic nervous system dysfunction, atherosclerosis, and mutations leading to deficient aldehyde dehydrogenase 2 (ALDH2) activity [11], etc. (Figure 2).

### 6.1. VSMCs Hypercontractility

Calcium and the calcium–CaM/MLCK/MLC pathway have a major involvement in VSMC contraction.

**Figure 3:**
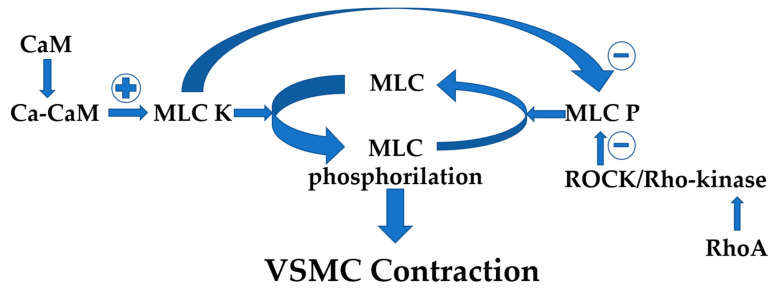
The mechanisms of VSMC contraction. Ca = calcium, CaM = calmodulin, MLC = myosin light chain, MLC K = myosin light chain kinase, MLC P = myosin light chain phosphatase; ROCK = Rho-associated coiled-coil kinase.

After calcium binding with calmodulin (CaM), the resultant calcium–CaM complex directly activates the myosin light chain (MLC) kinase (MLCK). MLC kinase induces MLC phosphorylation, resulting in VSMC contraction. On the contrary, under MLC phosphatase (MLCP), MLC is dephosphorylated [168]. An increase in MLC phosphorylation is obtained either via MLC kinase action, a kinase activated in response to increased free calcium levels [168], or via inhibition of MLC phosphatase, an enzyme inhibited by ROCK/Rho-kinase [169,170]. Through phosphorylation of the myosin-binding subunit of myosin phosphatase, Rho-kinase inactivates the phosphatase, raising MLC phosphorylation and contraction [169,171] (Figure 3).

It is considered that the Rho pathway plays a major role in CAS pathogenesis. Numerous molecules/situations determine VSMC hyper-response by activating the RhoA/RhoK pathway, such as oxidized low-density lipoprotein (oxLDL), ROS, inflammation, and chronic stress.

Rho, a small G-protein, modulates the calcium sensitization of VSMCs and acts by inhibiting myosin phosphatase activity [172,173]. Rho-kinase is an effector of Rho. The Rho/Rho-kinase pathway is substantially involved in the pathogenesis of cardiovascular disease [174]. More than this, some pleiotropic statin effects are mediated by their inhibitory effects on Rho and subsequent inhibition of Rho-kinase [175].

Overall, the expression of Rho-kinase is increased at the atherosclerotic and inflammatory level and leads to artery hypercontraction [176].

Some mutations associated with increased Rho-kinase activity were found at a higher incidence in patients with vasospastic angina as compared to controls, and were also more frequent in Japanese compared to Caucasian patients [177].

At the cellular level, Rho-kinase downregulates eNOS [178] and up-regulates tissue factor in the intima [179].

Activated Rho-kinase causes VSMC hypercontraction and enhances VSMC proliferation and migration while inhibiting VSMC apoptosis in the media [180].

Rho-kinase mediates the up-regulation of various pro-inflammatory molecules, thrombogenic molecules, fibrogenic molecules [113], and oxidative stress [181]. Rho-kinase downregulates endothelial nitric oxide synthase [178]. Rho-kinase activation is associated with a decrease in NO production and the acceleration of inflammation, thrombosis, and fibrosis.

Rho-kinase expression is stimulated by inflammatory stimuli, such as angiotensin II and IL-1 [167], and by remnant lipoproteins [182] in coronary VSMCs.

Rho-kinase increases the accumulation of inflammatory cells at the adventitia [183].

A large amount of data suggest that Rho-kinase plays a major role in coronary vasospasm. In experimental studies, it has been shown that intracoronary administration of an inhibitor of the Rho-kinase pathway such as fasudil/hydroxyfasudil [56,166] inhibits coronary spasm and MLC phosphorylations at the spastic coronary segments level [166].

One specific mechanism for vasoconstriction observed in CAS is represented by the increased activity of Rho-kinase [184] that leads to VSMC hyper-constriction [113,171]. Rho/ROCK activity is enhanced in coronary arteries in cases of decreased NO endothelial activity [185,186].

Besides promoting VSMC sensitivity to calcium, other effects are represented by the downregulation of NO synthase and VSMC proliferation [178,187].

More than this, Rho-kinase activity in circulating leukocytes is a diagnosis marker for CAS and correlates with disease activity [41,188]. One study [157] concluded that Rho-kinase activity in circulating leukocytes is significantly higher in patients with VSA than in those without coronary spasm, and that it has circadian variations only in patients with VSA, peaking around 6 AM. Another interesting conclusion of the same study was that coronary vasoconstriction in response to Ach is higher in patients with CAS than in those without vasospastic angina, correlating with Rho-kinase activity in circulating leukocytes at the moment of spasm inducing. Only in patients with VSA is Rho-kinase activity is positively correlated with basal coronary tone and parasympathetic nervous activity and negatively correlated with sympathetic activity.

Rho-kinase activation proved in peripheral leucocytes is associated with a decreased expression of NO synthase [178], and with an increased synthesis of VSMCs [189].

In humans and porcine models, it has been shown that hydroxyfasudil, a Rho-kinase inhibitor, prevents CAS [190,191]. Statins also block the RhoA/ROCK pathway [185,186], with antispastic effects.

Another contribution to VSMC contractility is attributed to K_ATP_ channel function, so dysfunction of K_ATP_ channels leads to the hypercontraction of smooth muscle cells, independent of the presence of atherosclerotic lesions [192,193].

It is considered that PLC and PKC are majorly involved in CAS pathogenesis based on increased PLC activity in the hyper-reactivity of coronary arteries and correlating with its magnitude. More than this, numerous molecules associated with VSMC hyper-contraction activate the PKC pathway [29]. It seems that the downstream PKC signals regulate VSMC contraction, with one of the most important downstream mediators being C-kinase potentiated protein phosphatase-1 inhibitor (CPI-17), which is involved in MLCP inactivation [194].

### 6.2. Endothelial Dysfunction

In essence, in the presence of an intact endothelial surface, exposure of VSMC to Ach results in vasodilation [88]. In conditions of either dysfunctional endothelium or increased sensitivity of VSMC, exposure to Ach determines coronary vasoconstriction [195].

In patients with CAS, other endothelium-dependent vasodilators besides Ach, such as ergonovine, histamine, and serotonin, induce coronary vasoconstriction instead of coronary vasodilation. The main issue is that dysfunctional NO synthase leads to the deficient release of NO, which triggers CAS [22,196,197]. Endothelial dysfunction alone cannot fully explain this phenomenon, but the interplay between endothelial dysfunction completes the understanding of the mechanisms of CAS [42]. A particular situation of interest is that not all patients with CAS exhibit endothelial dysfunction, which promotes the idea that it does not represent an essential physiopathological event for CAS [198].

However, ex vivo studies in rat and human arteries have shown that the main contribution of NO to vascular relaxation refers mainly to large arteries, such as coronary arteries; meanwhile, EDHF has a significant role in coronary microvessels [56,199,200].

NO deficiency is involved in VSA pathogeny, as is shown by the fact that eNOS T-786C mutation enhances the response to the constrictor effects of Ach, caused by a decrease in endothelial NO synthesis [201]. e-NOS polymorphism is reported in one-third of patients, mainly in Asians, but also in Caucasians [202].

High estrogen levels act as a protective factor through their capacity to enhance NO synthase activity [203]. This explains, at least partially, why CAS is more frequent in postmenopausal women [52].

One causal factor for NO degradation and endothelial dysfunction is represented by elevated oxidative stress [109], as is shown by the fact that antioxidants such as vitamin E or vitamin C can restore endothelial reactivity [204,205].

In patients with CAS, the levels of oxygen-reactive agents (such as thioredoxin, a cytoprotective enzyme against oxidative stress) [206] are high, and the levels of antioxidants (e.g., C and E vitamins) are low [207,208]. Vasoconstriction is a consequence of NO degradation by reactive oxygen species (ROS). In patients with CAS, the intracoronary injection of antioxidants such as vitamin C showed improvement in endothelial function [52].

Although data highlight the role of endothelial dysfunction in the pathophysiology of CAS, certain observations raise the question of whether the emphasis should be placed on the major contribution of endothelial dysfunction to the occurrence of CAS or rather on adventitial inflammation and VSMC hyper-reactivity. Thus, although endothelial dysfunction is diffuse, rather, systemic, epicardial coronary spasm is localized. Furthermore, it has been observed that vasodilation in response to P substance and bradykinin is preserved in spastic coronary segments in patients with variant angina [165]. The fact that VSMC hyper-reactivity is an important contributor to the pathophysiology of CAS is also suggested by the observation that nitrates remove the coronary spasm through VSMC relaxation, without affecting endothelial dysfunction [209].

In endothelial dysfunction, ET-1 levels are increased, especially in the microvasculature. ET-1 is expressed in VSMC and macrophages [83]. ET-1 circulating levels are increased in patients with CAS [83,210], contributing to the high vasomotor tone, an effect mediated by the ET-A receptor [211]. The increased ET-1 levels also increase protein kinase C activation, and therefore serotonin-induced vasoconstriction in the coronary vessels is increased [29].

High ET-1 levels enhance coronary contraction induced by serotonin [212] and also inhibit NO synthesis through the activation of protein kinase C [213]. High levels of ET-1 vascular receptors are observed in smokers, and the ET-1 level is increased after alcohol consumption [214,215].

### 6.3. Inflammation

A large body of evidence supports the thesis of chronic low-grade inflammation and CAS association. The pathophysiological link between inflammation and CAS is suggested by both experimental observations and post-mortem studies, which have highlighted inflammatory cells in spastic coronary segments [216,217].

Tobacco smoking, which represents the main risk factor for CAS, is also associated with chronic low-grade inflammation [218].

A number of inflammatory biomarkers and adhesive molecules are elevated in CAS, such as hs-CRP [22,83], IL- 6, soluble CD40 ligands [83], P-selectin [219], soluble intercellular adhesion molecule-1 (ICAM-1), and soluble vascular adhesion molecule-1 (VCAM-1) [220]. More than this, the C-reactive protein level correlates with other plasma inflammatory markers (such as IL-6) and also with CAS prognosis [22].

C-reactive protein (CRP), a systemic inflammatory mediator whose levels are associated with endothelial dysfunction, is a causal factor of CAD and a predictor of evolution in CAD alike. It decreases nitric oxide (NO) synthase and prostacyclin synthase expression, leading to an impaired vasodilator capacity of the endothelial surface [221,222]. hs-CRP is considered a marker of abnormal coronary reactivity in patients with non-obstructive coronary disease [223] and it correlates with mortality in patients with CAD [224].

Of all the inflammatory markers, the ones that correlate best with the presence of the spasm in the epicardial vessels and microvasculature [225] are CRP and sCD40L. Particularity in CAS, adventitial and PVAT inflammation are significant [225].

Inflammation plays a role in the activation of the ROCK pathway, particularly for certain cytokines such as TNF and IL-1 [226,227,228].

Increased PVAT inflammation has been evidenced using the CTA technique [229] and PET/CT scanning [225].

The increased vasa vasorum formation in the outermost layer of coronary arteries positively correlates with Rho-kinase activity in periphery leukocytes [32]. PVAT inflammation leads to vasoconstriction through a mechanism that involves the calcium sensitivity of coronary VSMC [230].

Endothelial microparticles are other components associated with vascular inflammation. Microparticles (MPs) are defined as membrane vesicles that originate from endothelial cells, smooth muscle cells, and circulating elements (leukocytes, platelets, and erythrocytes) [231]. They transport multiple molecules, from nucleic acid to proteins and lipids [232].

Endothelial MPs, a population that belongs to MPs, initiate endothelial dysfunction and are involved with various cardiovascular diseases, mainly atherosclerosis [233,234]. EMPs are regarded as emerging biomarkers of endothelial injury and dysfunction [235], and EMP levels are significantly higher in patients with cardiovascular disease than in healthy volunteers [236].

T-lymphocyte-derived MPs cause endothelial dysfunction in arteries and arterioles by influencing the NO and prostacyclin pathways [237]. There is evidence that MPs release inflammatory cytokines or chemokines and increase the expression of endothelial cell adhesion molecules [238,239,240]. Leukocyte-derived MPs show low levels in normal conditions but their expression significantly increases in systemic conditions and also in vascular inflammation [241,242].

MPs up-regulate endothelial release of pro-inflammatory mediators such as IL-6, monocyte chemoattractant protein-1 (MCP-1), inducible nitric oxide synthase (iNOS), and cyclooxygenase-2 (COX-2) [241]. It was shown in vitro that MPs can up-regulate E-selectin and determine the enhancement of endothelial gene expression and release of mediatory inflammation such as IL-6, IL-8, ICAM-1, and VCAM-1 [240].

Platelets may have a role in CAS pathogenesis, as long as activated platelets can induce IL-1β-dependent activation of endothelial cells [243].

Some in vitro and in vivo studies have shown hyper-aggregable platelets with decreased anti-aggregatory responses to NO in patients with CAS, not only in the acute phases of CAS but also in the chronic phase of the disease [244]. During acute symptomatic exacerbations, platelet resistance to NO is exacerbated, together with an increase in tryptase concentration (a marker of mast cell activation) and an increase in platelet MPs [245].

### 6.4. Autonomic Nervous System Unbalance

The circadian fluctuations in CAS incidence [22] suggest ANS involvement in the pathophysiology of CAS. Ach’s involvement in coronary spasm and the circadian changes in the distribution of CAS, particularly early in the morning, support the thesis of parasympathetic nervous system contribution to the coronary vasospasm [246]. As suggested by the increase in catecholamine level and adrenergic receptor activity, the sympathetic nervous system is also involved, with decreased parasympathetic activity after an ischemic attack [247].

In humans and non-human primates, the peak of vagal activity is recorded during the nighttime. Diminished circadian rhythms, especially with reduced or absent nighttime rise in vagal activity, correlate to a higher risk of CAS and other cardiovascular disorders [248]. Additionally, research indicates that individuals with diminished vagal activity and blunted diurnal variation in heart rate are at higher risk for cardiovascular diseases. This autonomic imbalance can lead to endothelial dysfunction, impaired coronary blow flow regulation, and increased vulnerability to ischemic episodes [249].

This circadian pattern of cardiovascular events overlaps with the higher sympathetic tone on awakening: higher catecholamine levels [250], and higher heart rate and blood pressure [251,252] in obstructive coronary disease. In addition, there is evidence that beta-blocker administration in the morning eliminates the morning peak in the incidence of myocardial infarction [253]. However, this does not apply to patients with vasospastic angina, as neither beta-blockers nor alpha-blockers have proven effective in controlling the symptoms of CAS. This is despite evidence which suggests that catecholamines and sympathetic stimuli can trigger CAS [22]. A study on the role of the parasympathetic nervous system in the development of CAS concluded that the vast majority of patients developed CAS along with associated symptoms when injecting acetylcholine into coronary arteries in patients diagnosed with variant angina [254].

Most often, CAS occurs at rest, between midnight and early morning [255], when the parasympathetic tone is elevated. The contribution of the parasympathetic system to CAS occurring in patients with VSA is also supported by the observation of spasms after intracoronary administration of Ach [256].

In untreated patients with myocardial ischemia, the analysis of 24 h ECG recordings has shown episodes of ST changes, suggesting MI is at least two or three times more frequent in the morning than at night between 6 AM and noon. The same vulnerability to myocardial ischemia was observed later in the day, between 6 and 8 PM, and an important thing to mention is that greater ST-segment displacements were observed when exercises were performed in the afternoon, rather than in the morning. A different circadian pattern was observed in variant angina, as the ST changes were more frequent at night, especially during the second half of sleep (between 2 and 4 AM) [257].

Research regarding heart rate variability (HRV) and norepinephrine release following insulin infusion in patients experiencing variant angina has indicated that elevated vagal tone and heightened sensitivity to adrenergic stimulation may initiate coronary artery spasm [258]. The analysis of HRV reflects the sympathovagal balance; some studies have shown that in CAS, it has a different pattern when compared to organic stenosis [259].

Autonomic nervous system dysfunction can be evaluated through a simple, non-invasive tool: heart rate recovery (HRR). It has two components: a fast component dictated by cardiac parasympathetic reactivation and a slow component dictated by sympathetic withdrawal [260]. HRR following exercise is becoming recognized as a significant prognostic indicator. Previous research has demonstrated that a blunted HRR, defined as a reduction in heart rate of less than 12 beats per minute from peak exercise to one minute into recovery, is a strong predictor of cardiovascular and overall mortality [260,261].

Kim H et al. found in their study that HRR was lower in CAS patients compared to non-CAS patients, with a significant association between the severity of spasm and HRR. Also, their study has shown that blunted HRR is an independent predictor of CAS presence [260].

Understanding the interplay between the autonomic nervous system and circadian rhythms is essential for developing effective strategies to manage and prevent CAS.

### 6.5. Interplay of Factors in the Physiopathology of CAS

All coronary artery layers are terminal-type vessels, and the expression of CAS risk factors results in endothelial dysfunction, VSMCs hyper-activity, and inflammation of the adventitia and perivascular adipose tissue [30].

At the core of CAS pathophysiology lies the abnormal contractility of VSMCs, which is driven by both calcium-dependent and calcium-independent pathways. The Rho pathway plays a crucial role here, as the RhoA/RhoK pathway is activated in various conditions, such as inflammation, chronic hypoxia, or chronic stress, and various elements may be involved in its expression [56,262].

Endothelial dysfunction and subsequent impaired NO production shift the balance in favor of vasoconstrictors and also amplify the aggregation of platelets and oxidative stress, leading to a vicious cycle of vasospasm episodes [263].

ROS plays an important role in the pathogenesis of CAS through its increase in the endothelial cells caused by various factors. This subsequently leads to NO conversion into peroxynitrite, which is an oxidizing substance [264]. ROS also exhibits pro-inflammatory effects alongside other peripheral blood elements [265]. Inflammation was demonstrated to be a major contributor to CAS, and a special role is played by histamine-releasing mast cells, alongside the perivascular tissue that enables the contraction of VSMCs [266,267,268]. A variety of inflammatory markers associated with CAS have been identified [37,269]. In conclusion, both inflammation and oxidative stress are key triggers for CAS.

The autonomic nervous system dysregulation represents a critical factor which exaggerates vasospastic responses in patients with increased sympathetic reactivity and lower vagal tone; the circadian patterns of CAS also support the role of autonomic fluctuations in the onset of vasospasm [270,271].

Ultimately, the multifactorial nature of CAS based on the interplay between VSMC hyperreactivity, endothelial dysfunction, inflammation, oxidative stress, and autonomic dysregulation prompts further investigation into the pathogenesis and requires a personalized approach to diagnosis and treatment (Table 2).

### 6.6. Future Directions in the Research of CAS

Despite the significant progress in the understanding of CAS, a deeper investigation of the molecular pathways is required to understand the complex interplay between the factors involved in its pathogenesis. Novel biomarkers that contribute to CAS should be sought, and advanced molecular profiling techniques can be applied to study the behavior of VSMCs to different stimuli [272,273].

The study of genetic and epigenetic factors could play a crucial role in revealing the role of these elements in the predisposition to CAS and how personalized therapeutic approaches can be developed [274]. Furthermore, the attentive investigation of systemic conditions such as inflammatory, infectious, or rheumatoid diseases could shed light on common immunological or vascular pathways that might lead to vascular hyperreactivity [275,276,277]. Recent data show that immune cell profiles, particularly blood lymphocytes, may be used as biomarkers for endothelial dysfunction and vascular reactivity [278,279].

Innovations in treatment strategies could include targeting Rho-kinase and endothelial dysfunction with the use of Rho-kinase inhibitors and endothelial stabilizers, which may be helpful in patients with recurrent spasm; substances like tetrahydrobiopterin and L-arginine modulate endothelial function and could be used as supplements to the conventional approach [280,281]. Drug delivery systems including 3D-printed biodegradable drug-eluting platforms may be designed to release vasodilators at the site of CAS, and this may permit minimizing systemic effects and increasing therapeutic efficiency [282,283,284,285]. Moreover, therapeutic interventions on ET-1 with antagonists of endothelin receptor could play a crucial role in tackling CAS. Specifically, bosentan administration was followed by rarer and less severe coronary spasms [286]. Novel and improved antagonists of ET-1 and its receptor could represent areas of further analysis and may hold great promise in the pharmaceutical management of CAS.

Finally, future CAS research will probably adopt an interdisciplinary approach based on the insights from various diseases where vascular dysfunction and endothelial instability are common hallmarks [287,288,289,290]. Integrating these various fields can help CAS management to evolve therapies with a more accurate and personalized approach.

## 7. Conclusions

Understanding of pathophysiology of CAS is important for establishing novel diagnostic and therapeutic approaches. Compared with atherosclerotic disease, CAS exhibits distinct patterns such as fixed-time angina or symptoms elicited by exposure to cold which affect the epicardial arteries and microvasculature alike.

The hyperreactivity of VSMCs seems to be the central mechanism underlying this condition. However, this heightened contractility is not an isolated event, but rather a response to multiple triggers, including endothelial dysfunction, oxidative stress, inflammation, and autonomic nervous system imbalances. Furthermore, understanding the pivotal role of VSMCs and their interactions with various pathophysiological factors is essential for developing targeted therapies to prevent and manage CAS.

The prophylactic treatment of CAS with L-channel calcium antagonists to decrease the vasoconstrictor response has only a limited symptomatic benefit, suggesting that inhibition of the Rho-kinase pathway might be a therapeutic alternative in CAS.

In conclusion, CAS is an under-recognized and distinct clinical entity that requires high precision and effective diagnostic and therapeutic approaches that go beyond the classical paradigms of ischemic heart disease. Therefore, future research is warranted to focus on personalized treatments targeting VSMC dysfunction and understanding the interplay with inflammation and the autonomic nervous system in order to improve patient care.

## Figures and Tables

**Table 1:** Diagnostic modalities for coronary artery spasm.

Diagnostic Modality	Key Features	Advantages	Limitations
Electrocardiography	Detection of transient ST-segment elevation/depression	Non-invasive Widely available Affordable	Limited sensitivity May not detect non-ST-elevation spasm
Exercise Stress Testing	Can trigger spasm in some patients Lacks specificity for CAS	Identifies exercise-induced ischemia Easy to perform	Low specificity for CAS Limited effectiveness
Holter Monitoring	Identifies transient ischemic changes over 24–48 h	Captures spontaneous spasms Useful in symptomatic patients	Requires patient compliance Limited event detection
Coronary Angiography with Spasm Provocation Test	Gold standard	Direct visualization of spasm High diagnostic accuracy	Invasive Risk of arrhythmia and other complications
Intravascular Ultrasound	Evaluates vessel wall morphology Detects atherosclerosis	Evaluates plaque burden and remodeling Useful in CAS suspicion	Limited ability to detect functional vasospasm
Optical Coherence Tomography	Provides high-resolution imaging of endothelial microstructure	Superior resolution to IVUS Study plaque burden and features	Limited availability Expensive Requires expertise
Cardiac Magnetic Resonance Imaging	Assesses myocardial perfusion and ischemia with vasodilator stress	Non-invasive Evaluates myocardial perfusion patterns	Lower spatial resolution compared to CT or angiography
Computed Tomography Angiography	Non-invasive Detects luminal irregularities and coronary anatomy	Non-invasive Detects anatomical abnormalities of the coronaries	Radiation exposure Limited functional assessment
Dual-Acquisition Cardiac CT	Offers functional and morphological vessel assessment with contrast imaging	Provides both anatomical and functional coronary evaluation	Requires contrast agent High radiation dose Cost-intensive
Positron Emission Tomography	Evaluates deficits in myocardial perfusion due to CAS	Functional assessment of blood flow in the myocardium	Requires radiotracers Expensive Limited availability

**Table 2:** Pathophysiological factors in coronary artery spasm.

Pathophysiological Factor	Mechanism of Action	Clinical Relevance
microparticles	↑ endothelial inflammation ↑ expression of E-selectin, ICAM-1, VCAM-1	potential biomarker for risk stratification target for novel anti-inflammatory therapies
endothelial dysfunction	↓ availability of NO ↑ expression of adhesion molecule	justifies use of endothelial-protective drugs (e.g., statins, ACE inhibitors, NO donors)
platelet activation	IL-1β-dependent endothelial activation impaired NO response hyperaggregability	supports the role of P2Y12 inhibitors, aspirin, and novel platelet-targeted therapies
inflammatory mediators	↑ inflammation ↑ vasoconstriction vascular remodeling	suggests IL-6, COX-2, and other inflammatory pathway inhibitors could improve CAS management
vascular smooth muscle hypercontractility	↑ vascular tone due (hypercontractility of smooth muscle cells)	provides rationale for vasodilators targeting vascular smooth muscle relaxation
Rho-kinase pathway activation	↑ sensitivity to vasoconstrictors impaired vascular relaxation	potential target for Rho-kinase inhibitors in CAS treatment
myosin light chain phosphorylation	↑ vasoconstriction increased spasm severity	correlates with CAS severity may predict recurrent vasospastic events
mast cell activation	↑ vascular hyperreactivity endothelial dysfunction	potential role for mast cell stabilizers as ancillary treatment of CAS
mild atherosclerotic lesions	predisposition for vasospasm by altering vascular reactivity	lipid-lowering therapies may reduce susceptibility for CAS in mild atherosclerosis
serotonin/histamine sensitivity	↑ vasospastic responses (in segments with mild atherosclerosis)	potential therapeutic targets in serotonin pathway

↑ increase/stimulate; ↓ decrease/inhibit.

## Data Availability

The data presented in this study are available on reasonable request from the corresponding author.

## Abbreviations

The following abbreviations are used in this manuscript:

Ach	Acetylcholine
ACS	Acute coronary syndrome
ALDH2	Aldehyde dehydrogenase 2
ANS	Autonomic nervous system
ATP-CFR	Adenosine triphosphate-induced coronary flow reserve
AV	Atrioventricular
CAD	Coronary artery disease
CaM	Calmodulin
CAS	Coronary artery spasm
CCT	Cardiac computed tomography
cMRI	Cardiac magnetic resonance imaging
COX-2	Cyclooxygenase-2
CPI-17	C-kinase-activated protein phosphatase-1 inhibitor of 17 kDa
CRP	C-reactive protein
CSFP	Coronary slow flow phenomenon
CTA	Computed tomography angiography
EC	Endothelial cells
ECG	Electrocardiogram
ECM	Extracellular matrix
EDHF	Endothelium-derived hyperpolarization factor
EMPs	Endothelial microparticles
eNOS	Endothelial nitric oxide synthase
EPCR	Endothelial cell protein C receptor
ET-1	Endothelin-1
FFR	Fractional flow reserve
HRR	Heart rate recovery
HRV	Heart rate variability
hs-CRP	High-sensitivity C-reactive protein
ICAM-1	Intercellular adhesion molecule-1
IL-1	Interleukin-1
IL-6	Interleukin-6
IL-8	Interleukin-8
INOCA	Ischemia with non-obstructive coronary arteries
iNOS	Inducible nitric oxide synthase
IV ER	Intravenous ergonovine
IVUS	Intravascular ultrasound
LAD	Left anterior descending
LDL	Low-density lipoproteins
LGE	Late gadolinium enhancement
MB	Myocardial bridging
MCP-1	Monocyte chemoattractant protein-1
MI	Myocardial ischemia
MINOCA	Myocardial infarction with non-obstructive coronary arteries
MLC	Myosin light chain
MLCK	Myosin light chain kinase
MLCP	Myosin light chain phosphatase
MPs	Microparticles
NO	Nitric oxide
OCT	Optical coherence tomography
oxLDL	Oxidized low density lipoprotein
PCI	Percutaneous coronary intervention
PET/CT	Positron emission tomography/computed tomography
PKC	Protein kinase C
PLC	Phospholipase C
PVAT	Perivascular adipose tissue
ROCK	Rho-associated coiled-coil-containing protein kinase
ROS	Reactive oxygen species
sCD40L	Soluble CD40 ligand
TNF	Tumor necrosis factor
TXA2	Thromboxane A2
VCAM-1	Vascular adhesion molecule-1
VSA	Vasospastic angina
VSMC	Vascular smooth muscle cell
